# Class II transactivator induces expression of MHC-I and MHC-II in transmissible Tasmanian devil facial tumours

**DOI:** 10.1098/rsob.220208

**Published:** 2022-10-19

**Authors:** Chrissie E. B. Ong, Yuanyuan Cheng, Hannah V. Siddle, A. Bruce Lyons, Gregory M. Woods, Andrew S. Flies

**Affiliations:** ^1^ Menzies Institute for Medical Research, College of Health and Medicine, University of Tasmania, Private Bag 23, Hobart, TAS 7000, Australia; ^2^ School of Life and Environmental Sciences, The University of Sydney, Sydney, NSW 2006, Australia; ^3^ Department of Biological Sciences, University of Southampton, Southampton SO17 1BJ, UK; ^4^ Institute for Life Sciences, University of Southampton, Southampton SO17 1BJ, UK; ^5^ Tasmanian School of Medicine, College of Health and Medicine, University of Tasmania, Hobart, TAS 7005, Australia

**Keywords:** transmissible cancer, devil facial tumour, DFTD, MHC, CIITA

## Abstract

MHC-I and MHC-II molecules are critical components of antigen presentation and T cell immunity to pathogens and cancer. The two monoclonal transmissible devil facial tumours (DFT1, DFT2) exploit MHC-I pathways to overcome immunological anti-tumour and allogeneic barriers. This exploitation underpins the ongoing transmission of DFT cells across the wild Tasmanian devil population. We have previously shown that the overexpression of NLRC5 in DFT1 and DFT2 cells can regulate components of the MHC-I pathway but not MHC-II, establishing the stable upregulation of MHC-I on the cell surface. As MHC-II molecules are crucial for CD4^+^ T cell activation, MHC-II expression in tumour cells is beginning to gain traction in the field of immunotherapy and cancer vaccines. The overexpression of Class II transactivator in transfected DFT1 and DFT2 cells induced the transcription of several genes of the MHC-I and MHC-II pathways. This was further supported by the upregulation of MHC-I protein on DFT1 and DFT2 cells, but interestingly MHC-II protein was upregulated only in DFT1 cells. This new insight into the regulation of MHC-I and MHC-II pathways in cells that naturally overcome allogeneic barriers can inform vaccine, immunotherapy and tissue transplant strategies for human and veterinary medicine.

## Graphical abstract

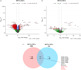

## 1. Introduction

The Tasmanian devil is the largest extant carnivorous marsupial and is endemic to the island state of Tasmania. Following the emergence of devil facial tumour disease (DFTD) in 1996, the population of devils has declined precipitously, threatening the persistence of devils in the wild [1]. DFTD is caused by two independent transmissible cancers of Schwann cell origin, referred herein as DFT1 and DFT2 [2,3]. DFT1 was discovered northeast of Tasmania in 1996 while the second tumour, DFT2, was found in 2014 in the D'Entrecasteaux channel, southeast Tasmania. Both tumour types are clonal cell lines that harbour distinct genetic profiles differing from individual host devils [2,3]. DFT cells are transmitted as a malignant allograft among devils through social interactions.

Genetic differences between host and tumour, particularly at the major histocompatibility complex (MHC) loci [4], should induce immune-mediated allograft rejection. However, the 25 years of ongoing DFT1 transmission suggests that DFT1 cells have evolved to evade immune defences. The lack of anti-DFT immune responses has predominantly focused on the loss of MHC-I from the surface of DFT1 cells. This occurs via epigenetic downregulation of several components of the MHC-I antigen processing pathway [5] and a hemizygous deletion of beta-2 microglobulin (B2M), which is necessary for stabilizing MHC-I complexes on the cell surface [6]. Natural and immunotherapy-induced tumour regressions have been observed in devils, along with antibody responses to DFT1 cells, albeit primarily in the context of MHC-I [7–9]. Conversely, the emerging DFT2 tumours do express MHC-I [10], suggesting that other immune evasion mechanisms are important.

Given the role of MHC-I in antigen display and anti-DFT humoral response, the manipulation of MHC-I expression on DFT cells is an attractive target to improve host responses towards DFT cells and mitigate the effects of disease in the wild devil population. An upregulation of MHC-I on DFT cells should enhance MHC-I-restricted tumour-specific cytotoxic CD8^+^ T cell response. However, this approach alone proved to be insufficient for eliciting protective immunity, as exemplified in immunization trials of naïve devils against DFT1 [9]. Although CD8^+^ T cells are recognized as the major effector cells in tumour elimination, CD4^+^ T cell help is critical in facilitating an effective anti-tumour immune response. CD4^+^ helper T cells play a multifaceted role of orchestrating the cellular and humoral immune response. From cytokine production to the expression of co-stimulatory molecules, CD4^+^ helper T cells initiate, augment and sustain the effector function of not only CD8^+^ T cells and B cells but also innate cells [11–14]. Moreover, there are evidence of CD4^+^ T cells initiating allograft rejection independently of CD8^+^ T cells in mice [15,16].

The activation of CD4^+^ T cells involves recognition of antigens presented on MHC-II complexes. In contrast with MHC-I, constitutive expression of MHC-II is restricted to thymic epithelial cells, activated human T cells and professional antigen-presenting cells (APCs) such as B cells, dendritic cells and macrophages. However, *de novo* MHC-II expression can be induced in non-haematopoietic cells including tumour cells by the inflammatory cytokine interferon gamma (IFNG) [17]. The expression of MHC-II proteins on DFT cells could complement the anti-tumour response to tumour MHC-I in a DFT vaccine for immunization and immunotherapy. Both constitutive and IFNG-induced expressions of MHC-II genes are mediated by the Class II transactivator (CIITA), making it the master regulator of MHC-II expression [18,19]. CIITA functions as a transcriptional co-activator that recruits transcription factors of the MHC enhanceosome to the SXY module for transcription of MHC-II genes and *CD74* [20–23]. The SXY module is also found in the promoters of MHC-I genes [24]; therefore, CIITA is capable of modulating the expression of MHC-I, particularly in cell lines with low to no MHC-I expression [25,26].

The presence of MHC-II molecules in DFT cells has not been described, although CIITA and some MHC-II transcripts can be upregulated *in vitro* in DFT1 cells with IFNG treatment [5]. We have previously genetically modified DFT1 and DFT2 cells that overexpress the MHC-I transactivator NLRC5 to induce stable expression of MHC-I on the cell surface [8]. The lack of MHC-II expression in DFT cells provided an opportunity to conduct similar investigations into the role of CIITA in MHC-II regulation in Tasmanian devils and transmissible cancers. Transcriptomic and protein-based analyses showed that CIITA upregulates the expression of genes associated with MHC-I and MHC-II antigen processing and presentation in DFT cells. The ability to modulate antigen presentation in transmissible cancer cells in the context of MHC uncovers additional targets for anti-tumour immune response and the potential for recruitment of CD4^+^ T cell help.

## 2. Materials and methods

### 2.1. Cells and cell culture conditions

Cell lines that were used in this study include DFT1 cell line C5065 strain 3 [27] (RRID:CVCL_LB79), and DFT2 cell lines: RV (RRID:CVCL_LB80) and JV (RRID:CVCL_A1TN) [3] (table 1). DFT1 C5065 was provided by A-M Pearse and K. Swift of the Department of Primary Industries, Parks, Water and Environment (DPIPWE) (Hobart, TAS, Australia) and was previously established from DFT1 biopsies obtained under the approval of the Animal Ethics Committee of the Tasmanian Parks and Wildlife Service (permit numbers A0017090 and A0017550) [27]. DFT2 cell lines RV and JV were established from single-cell suspensions obtained from tumour biopsies [3]. Cells were cultured at 35°C with 5% CO_2_ in Gibco RPMI 1640 medium with L-glutamine (Thermo Fisher Scientific, Waltham, MA, USA) supplemented with 10% heat-inactivated fetal bovine serum (Bovogen Biologicals, Melbourne, VIC, Australia), 1% (v/v) Gibco Antibiotic-Antimycotic (100X) (Thermo Fisher Scientific), 10 mM Gibco HEPES (Thermo Fisher Scientific) and 50 µM 2-mercaptoethanol (Sigma-Aldrich, St. Louis, MO, USA) (complete RPMI medium).

**Table 1.** List of all DFT cell lines and treatments.

ID no.	sample name	parent cell line	treatment	references	ENA project no.
1	DFT1.WT	DFT1 C5065	untreated	Patchett *et al*. [28]	PRJNA416378
2	DFT2.WT^RV^	DFT2 RV	untreated	Patchett *et al*. [29]	PRJEB28680
3	DFT2.WT	DFT2 JV	untreated	Ong *et al*. [8]	PRJEB39847
4	DFT1.WT + IFNG	DFT1 C5065	5 ng mL^−1^ IFNG, 24 h	Ong *et al*. [8]	PRJEB39847
5	DFT2.WT^RV^+IFNG	DFT2 RV	5 ng mL^−1^ IFNG, 24 h	Ong *et al*. [8]	PRJEB39847
6	DFT1.BFP	DFT1 C5065	transfected with empty vector pSBbi-BH	Ong *et al*. [8]	PRJEB39847
7	DFT2.BFP	DFT2 JV	transfected with empty vector pSBbi-BH	Ong *et al*. [8]	PRJEB39847
8	DFT1.NLRC5	DFT1 C5065	transfected with NLRC5 vector pCO1	Ong *et al*. [8]	PRJEB39847
9	DFT2.NLRC5	DFT2 JV	transfected with NLRC5 vector pCO1	Ong *et al*. [8]	PRJEB39847
10	DFT1.CIITA	DFT1 C5065	transfected with CIITA vector pCO2	this study	PRJEB45867
11	DFT2.CIITA	DFT2 JV	transfected with CIITA vector pCO2	this study	PRJEB45867

### 2.2. Plasmid construction

The coding sequence for full-length devil *CIITA* (XM_023497584.2) was isolated from cDNA of devil peripheral blood mononuclear cells by PCR using Q5 Hotstart High-Fidelity 2X Master Mix (New England Biolabs (NEB), Ipswich, MA, USA) (see electronic supplementary material, table S1 for list of primers and reaction conditions). Sleeping Beauty (SB) transposon plasmid pSBbi-BH [30] (a gift from Eric Kowarz; Addgene no. 60515, Cambridge, MA, USA) was digested at SfiI sites (NEB) with the addition of Antarctic Phosphatase (NEB) to prevent re-ligation. Devil *CIITA* was then cloned into SfiI-digested pSBbi-BH using NEBuilder HiFi DNA Assembly Cloning Kit (NEB). The assembled plasmid pCO2 was transformed into NEB 5-alpha competent *Escherichia coli* (high efficiency) (NEB) according to manufacturer's instructions (see electronic supplementary material, figure S1 for plasmid maps). Positive clones were identified by colony PCR, and the plasmids were isolated using NucleoSpin Plasmid EasyPure kit (Macherey-Nagel, Düren, Germany). The DNA sequence of the cloned devil *CIITA* transcript was verified by Sanger sequencing using Big Dye Terminator v3.1 Cycle Sequencing Kit (Applied Biosystems (ABI), Foster City, CA, USA) and Agencourt CleanSEQ (Beckman Coulter, Brea, CA, USA) per manufacturer's instructions. The sequences were analysed on 3500xL Genetic Analyzer (ABI) (see electronic supplementary material, table S2 for list of sequencing primers). For detailed step-by-step protocols for plasmid design and construction, reagent recipes and generation of stable cell lines, see Bio-protocol no. e3696 [31,32].

### 2.3. Transfection and generation of stable cell lines

DFT1 and DFT2 cell line C5065 and JV, respectively, were transfected with plasmid pCO2 to generate stable cell lines that overexpress CIITA. DNA transfections were performed using polyethylenimine (PEI) (1 mg ml^−1^, linear, 25 kDa; Polysciences, Warrington, FL, USA) at a 3 : 1 ratio of PEI to DNA (w/w) as previously described [8]. Briefly, DFT cells were co-transfected with pCO2 and SB transposase vector pCMV(CAT)T7-SB100 [33] (a gift from Zsuzsanna Izsvak; Addgene plasmid no. 34879) at a ratio of 3 : 1 in µg, respectively. One microgram of total plasmid DNA was used per millilitre of culture volume. The cells were incubated with the transfection solution overnight at 35°C with 5% CO_2_. The media was removed and replaced with fresh complete RPMI medium. Forty-eight hours of post-transfection, the cells were observed for expression of reporter gene mTagBFP. Positively transfected cells were selected with 1 mg ml^−1^ hygromycin B (Sigma-Aldrich) for 7 days before being maintained in 200 µg ml^−1^ hygromycin B in complete RPMI medium. The two tumour cell lines were also transfected with empty vector pSBbi-BH as controls.

### 2.4. RNA sequencing and analysis

RNA libraries were prepared, sequenced and processed as previously described [8,28,29]. Table 1 shows the source of RNA samples used in this study. Briefly, RNA extraction (two replicates per cell line) was performed using the Nucleospin RNA Plus Kit (Macherey-Nagel) following the manufacturer's instructions. mRNA libraries were prepared and sequenced at the Ramaciotti Centre for Genomics (Sydney, NSW, Australia). All RNA samples had RNA Integrity Number (RIN) scores of 10.0. Libraries were prepared using TruSeq Stranded mRNA Library Prep (Illumina Inc., San Diego, CA, USA) and single-end, 100-base pair sequencing was performed on an Illumina NovaSeq 6000 platform (Illumina). The quality of the sequencing reads was assessed using FastQC version 0.11.9 [34]. Raw FASTQ files for DFT1.CIITA and DFT2.CIITA have been deposited to the European Nucleotide Archive (ENA) and are available at BioProject no. PRJEB45867.

Subread version 2.0.0 [35] was used to align sequencing reads to the Tasmanian devil reference genome (GCA_902635505.1 mSarHar1.11) [36] and the number of reads mapped to a gene was counted using featureCounts [37]. The analysis of differentially expressed genes was performed using the statistical software R studio [38] on R v.4.0.0 [39]. Genes with less than 100 aligned reads across all samples were excluded from the analysis and raw library sizes were scaled using *calcNormFactors* in edgeR [40–42]. To account for varying sequencing depths between lanes, read counts were normalized by upper quartile normalization using *betweenLaneNormalization* in EDASeq [43,44]. Gene length-related biases were normalized by scaling read counts to transcripts per kilobase million (TPM). Differential expression analysis was carried out using the *voom* [45] function in *limma* [46] with linear modelling and empirical Bayes moderation [47]. To isolate differentially expressed genes, gene expression of CIITA- or NLRC5-expressing cell lines (DFT.CIITA, DFT.NLRC5) was compared against vector-only control (DFT.BFP) while IFNG-treated cells (DFT.WT + IFNG) was compared against untreated cells (DFT.WT), according to their respective tumour origin. Genes were defined as significantly differentially expressed by applying false discovery rate (FDR) < 0.05, and log_2_ fold change (FC) ≥ 2.0 (upregulated) or ≤ −2.0 (downregulated) thresholds (see electronic supplementary material, table S3 for list of differentially expressed genes). Scripts for RNA data processing and differential gene expression analysis are provided in the electronic supplementary material, methods S1.

Volcano plots and Venn diagrams of differentially expressed genes were created using EnhancedVolcano and Venny version 2.1, respectively [48,49]. Heatmaps were created from log_2_(TPM) values using the ComplexHeatmap [50] package in R studio. For functional enrichment analysis, over-representation of gene ontology (GO) biological processes in the list of differentially expressed genes was performed using Database for Annotation, Visualization and Integrated Discovery (DAVID) functional annotation tool [51,52]. The Tasmanian devil *Sarcophilus harrisii* was applied as the species for gene lists and background. Significant GO terms (GOTERM_BP_ALL) were selected by applying the following thresholds: *p*-value < 0.05 and FDR < 0.05. GO terms were sorted in descending order of fold enrichment values.

To simplify the identification of devil MHC allotypes and maintain consistency in nomenclature to previous works, MHC transcripts in this manuscript were renamed according to Cheng *et al*. [53] based on sequence similarity (see electronic supplementary material, table S4 for corresponding NCBI gene symbols). MHC transcripts *LOC100918485* and *LOC100918744*, which have not been previously characterized, are predicted to encode beta chains of the MHC-II DA gene based on gene homology. These transcripts were renamed as *SAHA-DAB_X1* and *SAHA-DAB_X2*, respectively. Similarly, genes without an official gene symbol (LOC prefixes) were given aliases based on the gene description on NCBI.

### 2.5. Flow cytometric analysis of B2M and MHC-II expression

Cultured cells were harvested using TrypLE Express Enzyme (1X) (Thermo Fisher Scientific) and counted using a haemocytometer. 1 × 10^5^ cells per well were aliquoted into round-bottom 96-well plates and washed with 1 X PBS (Thermo Fisher Scientific). Washing steps include centrifugation at 500 *g* for 3 min at 4°C to pellet cells before removal of supernatant. Cells were first stained with Invitrogen LIVE/DEAD Fixable Near-IR Dead Cell Stain kit (Thermo Fisher Scientific) diluted according to manufacturer's instructions for 30 min on ice, protected from light. After staining, cells were washed twice with 1X PBS. For MHC-II expression, a monoclonal mouse antibody against the intracellular tail of human HLA-DR *α* chain was used (Clone TAL.1B5, no. M0746, Agilent, Santa Clara, CA, USA). The detection of MHC-I on the surface of cells was performed using a monoclonal mouse antibody against devil B2M in supernatant (Clone 13-34-45; a gift from Hannah Siddle [5]). Cells for intracellular staining of HLA-DR were first fixed and permeabilized using BD Cytofix/Cytoperm Plus Fixation/Permeabilization Kit (BD Biosciences, North Ryde, NSW, Australia). All intracellular antibody staining, and washes were carried out in 1X BD Perm/Wash Buffer (BD Biosciences) while FACS buffer (PBS with 0.5% BSA, 0.02% sodium azide) was used for surface antibody staining. All cells were incubated with 1% normal goat serum (Thermo Fisher Scientific) for blocking, 10 min on ice. After that, cells were washed and incubated with either anti-human HLA-DR*α* (0.48 µg ml^−1^) or anti-devil B2M antibody (1 : 250 v/v dilution) for 30 min on ice. Cells were washed once and stained with goat anti-mouse IgG-Alexa Fluor 488 (2 µg ml^−1^, no. A11029, Thermo Fisher Scientific) for 30 min on ice, in the dark. Two final washes were given to remove excess secondary antibody. Fixed cells were resuspended in FACS buffer while the rest were resuspended in FACS fix solution (0.02% sodium azide, 1.0% glucose and 0.4% formaldehyde). Analysis was carried out using Cytek Aurora (Cytek Biosciences, Fremont, CA, USA). As a positive control for MHC-I expression, DFT cells were treated with 10 ng ml^−1^ devil recombinant IFNG [54] for 24 h.

### 2.6. Protein extraction and western blot

Cells were harvested and centrifuged at 500 *g* for 5 min at room temperature. The pellet was washed twice with cold 1 X PBS and weighed. Total cell protein was extracted by adding 1 ml RIPA Lysis and Extraction Buffer (Thermo Fisher Scientific), 10 µl Halt Protease Inhibitor Cocktail (Thermo Fisher Scientific) and 10 µl Halt Phosphatase Inhibitor Cocktail (Thermo Fisher Scientific) per 40 mg of wet cell pellet. The suspension was sonicated for 30 s with 50% pulse and then mixed gently for 15 min on ice. The mixture was centrifuged at 14 000 *g* for 15 min to pellet the cell debris. The supernatant was transferred to a new tube and total protein was quantified using EZQ Protein Quantitation kit (Invitrogen) according to manufacturer's instructions. Two replicates per cell line were prepared for protein extraction.

Twenty micrograms of protein per sample was used for target protein detection by western blot. Protein samples were subjected to SDS-PAGE using Bolt 4–12%, Bis-Tris, 1.0 mm Mini Protein Gel (Thermo Fisher Scientific). Briefly, protein samples were treated with 1X Bolt LDS Sample Buffer (Thermo Fisher Scientific) and 1X Bolt Reducing Agent (Thermo Fisher Scientific) at 70°C for 10 min. Samples were loaded onto the gel and run with 1 X Bolt MES SDS Running Buffer (Thermo Fisher Scientific) in the Mini Gel Tank (Thermo Fisher Scientific) at 100 V for 5 min followed by 200 V for 15 min. SeeBlue Plus2 Pre-stained Protein Standard (Thermo Fisher Scientific) was used as a molecular weight marker. Proteins were transferred to a nitrocellulose membrane using iBlot Transfer Stack, nitrocellulose, mini (Thermo Fisher Scientific) and iBlot Gel Transfer Device (Thermo Fisher Scientific) using the following settings: 20 V for 7.5 min.

For immunodetection, the membrane was blocked with TBSTM (Tris-buffered saline (TBS): 50 mM Tris-HCl, 150 mM NaCl, pH 7.6), 0.05% Tween 20 and 5% skim milk) for 1 h at room temperature and rinsed twice with TBST (TBS, 0.05% Tween 20). Then, the membrane was incubated with: (i) rabbit polyclonal anti-beta actin antibody (no. ab8227, Abcam, Cambridge, UK) diluted in TBSTM (400 ng ml^−1^), (ii) mouse monoclonal anti-devil SAHA-UA/UB/UC in supernatant (Clone 15-25-18; a gift from Hannah Siddle [10]) or (iii) mouse monoclonal anti-devil SAHA-UK in supernatant (Clone 15-29-1; a gift from Hannah Siddle [10]) overnight at 4°C. The membranes were washed four times with TBST for a duration of 5 min each wash. After that, the membranes were incubated with either HRP-conjugated goat anti-mouse (250 ng ml^−1^; no. P0447, Agilent) or HRP-conjugated goat anti-rabbit immunoglobulin (62.5 ng ml^−1^; no. P0448, Agilent) diluted in TBSTM for 1 h at room temperature. The membranes were given final washes as described above. All incubation and washing steps were performed under agitation. Target protein expression was detected using Immobilon Western Chemiluminescent HRP Substrate (Merck Millipore, Burlington, MA, USA) according to manufacturer's protocol. Protein bands were visualized using Amersham Imager 600 (GE Healthcare Life Sciences, Malborough, MA, USA).

### 2.7. Flow cytometric analysis of serum antibody binding

Serum samples from four devils (My, TD4, TD5 and TD6), collected before (pre-immune) and after DFT1 clinical manifestations (immune), were used to assess antibody responses towards CIITA-expressing DFT cell lines (electronic supplementary material, table S5). The serum samples were identified as immune from the presence of anti-DFT1 antibodies, which were found to be predominantly against MHC-I on DFT1 cells [8]. ‘My’ was a devil that was immunized, challenged with DFT1 cells and subsequently treated with an experimental immunotherapy that induced tumour regression [9]. TD4, TD5 and TD6 were naturally DFT1-infected wild devils with either spontaneous tumour regressions (TD4); MHC-II^+^ and CD3^+^ tumour-infiltrating lymphocytes in the tumour (TD5) or B2M^+^ DFT1 cells in fine needle aspirations of a tumour (TD6) [7]. A devil with no clinical signs of DFTD during serum collection (TD7) was included as a negative control for antibody binding towards the DFT cell lines.

Cells were harvested and aliquoted into round-bottom 96-well plates as indicated above. After washing with PBS, cells were stained with LIVE/DEAD Fixable Near-IR Dead Cell Stain for 30 min on ice and washed twice with PBS. For blocking, cells were incubated with 1% normal goat serum for 10 min and washed once with FACS buffer. Serum samples were thawed on ice and diluted with FACS buffer (1 : 50 v/v). 50 µl of serum was added to cells for 1 h and then washed. After that, cells were stained with 10 µg ml^−1^ monoclonal mouse anti-devil IgG antibody (A4-D1-2-1, provided by WEHI) [55] diluted in FACS buffer for 30 min. The cells were washed and stained with 2 µg ml^−1^ goat anti-mouse IgG-Alexa Fluor 647 (no. A21235, Thermo Fisher Scientific) in FACS buffer for 30 min. After washing, cells were fixed in FACS fix solution and analysed on Cytek Aurora. All washing steps include two washes with FACS buffer unless indicated otherwise and all staining steps were carried out on ice, protected from light.

## 3. Results

### 3.1. Class II transactivator plays a dominant role in antigen presentation

**Figure 1.**
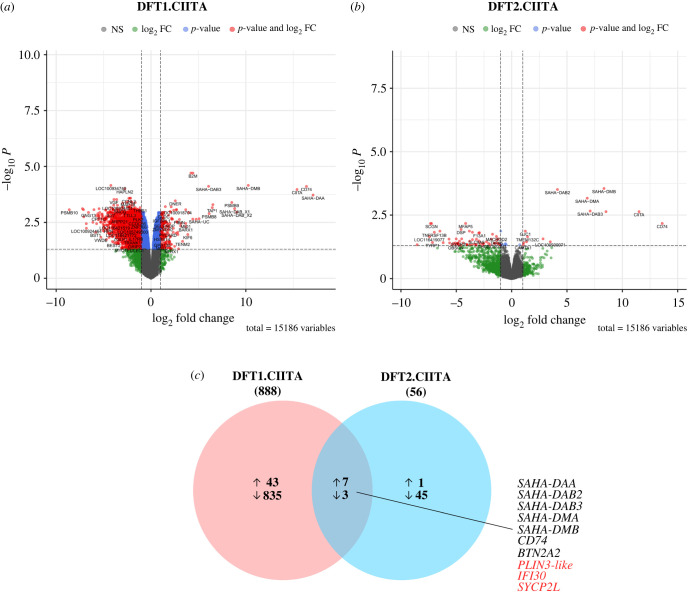
Volcano plots and Venn diagram of DEGs in DFT1 and DFT2 cells with CIITA overexpression. Volcano plots of DEGs in DFT1.CIITA (*a*) and DFT2.CIITA (*b*) with |log_2_FC| ≥ 2 and adjusted *p*-value (FDR) < 0.05. Change in gene expression was identified between DFT.CIITA and vector-only control DFT.BFP. (*c*) Venn diagram showing the number of mutually inclusive and exclusive upregulated (↑) and downregulated genes (↓) between DFT1.CIITA and DFT2.CIITA. The total number of DEGs is indicated in parenthesis under sample name. Mutually inclusive genes that were upregulated are indicated in black and downregulated in red.

To delineate the role of CIITA in DFT cells, differentially expressed genes following stable expression of CIITA were analysed by GO functional enrichment analysis. The transcriptome landscape of differentially expressed genes (|log_2_FC| ≥ 2, FDR < 0.05) in DFT1.CIITA and DFT2.CIITA are shown in figure 1*a*,*b*. Differential expression analysis revealed 888 genes, excluding CIITA, that were modulated in DFT1.CIITA compared to vector-only cell line DFT1.BFP (figure 1*c*; electronic supplementary material, table S3). In DFT2.CIITA, there were 56 genes that were differentially expressed relative to DFT2.BFP. Ten genes were commonly up- or downregulated by CIITA in DFT1 and DFT2 cells. Most of these genes were of the MHC-II antigen processing and presentation pathway. *SAHA-DAA, SAHA-DAB2* and *SAHA-DAB3* are devil classical MHC-II genes while *SAHA-DMA* and *SAHA-DMB* encode non-classical MHC-II. Others include *CD74*, butyrophilin subfamily 2 member A2 (*BTN2A2*) and gamma-interferon-inducible lysosomal thiol reductase (*IFI30*), which encode the invariant chain, an MHC-II chaperone, a T cell immunomodulatory molecule and an enzyme for lysosomal degradation of proteins, respectively. Except for *BTN2A2* and *IFI30*, these genes were among the most highly upregulated genes in the transcriptome of DFT1.CIITA (table 2) and DFT2.CIITA (table 3).

**Table 2.** Top 20 most significantly upregulated genes in DFT1.CIITA. See electronic supplementary material, table S3 for full list of differentially expressed genes and log2TPM values.

gene	gene description	MHC pathway	log_2_FC	FDR
*SAHA-DAA*	Class II histocompatibility antigen, DA alpha chain	Class II	17.09	1.90 × 10^−04^
*CD74*	CD74 molecule	Class II	16.39	7.77 × 10^−05^
*CIITA*	Class II MHC transactivator	Class II	15.40	1.07 × 10^−04^
*SAHA-DMB*	Class II histocompatibility antigen, DM beta chain	Class II	10.26	7.00 × 10^−05^
*SAHA-DAB_X2*	Class II histocompatibility antigen, DA beta chain	Class II	9.04	1.11 × 10^−03^
*SAHA-DAB_X1*	Class II histocompatibility antigen, DA beta chain	Class II	8.82	7.77 × 10^−04^
*PSMB9*	proteasome 20S subunit beta 9	Class I	8.52	4.08 × 10^−04^
*SAHA-DMA*	Class II histocompatibility antigen, DM alpha chain	Class II	6.52	5.12 × 10^−04^
*TAP1*	transporter 1, ATP binding cassette subfamily B member	Class I	6.46	6.95 × 10^−04^
*PSMB8*	proteasome 20S subunit beta 8	Class I	6.13	1.29 × 10^−03^
*SAHA-DAB3*	Class II histocompatibility antigen, DA beta chain	Class II	6.08	7.72 × 10^−05^
*SAHA-UC*	Class I histocompatibility antigen heavy chain	Class I	5.08	2.17 × 10^−03^
*SAHA-UA*	Class I histocompatibility antigen heavy chain	Class I	4.77	2.29 × 10^−03^
*B2M*	B2M	Class I	4.43	1.96 × 10^−05^
*SAHA-UB*	Class I histocompatibility antigen heavy chain	Class I	4.41	2.25 × 10^−03^
*SAHA-DAB2*	Class II histocompatibility antigen, DA beta chain	Class II	4.21	1.96 × 10^−05^
*ICOSLG*	inducible T Cell costimulator (ICOS) ligand	unrelated	3.98	1.14 × 10^−03^
*KIF6*	kinesin family member 6	unrelated	3.88	1.10 × 10^−02^
*BARX1*	BARX homeobox 1	unrelated	3.68	4.99 × 10^−03^
*MID1*	midline 1	unrelated	3.67	3.52 × 10^−03^

**Table 3.** Significantly upregulated genes in DFT2.CIITA. See electronic supplementary material, table S3 for full list of differentially expressed genes and log2TPM values.

gene	gene description	MHC pathway	log_2_FC	FDR
*CD74*	CD74 molecule	Class II	13.60	6.69 × 10^−03^
*CIITA*	Class II MHC transactivator	Class II	11.50	2.31 × 10^−03^
*SAHA-DAA*	Class II histocompatibility antigen, DA alpha chain	Class II	8.52	2.31 × 10^−03^
*SAHA-DMB*	Class II histocompatibility antigen, DM beta chain	Class II	8.34	2.81 × 10^−04^
*SAHA-DAB3*	Class II histocompatibility antigen, DA beta chain	Class II	7.09	2.19 × 10^−03^
*SAHA-DMA*	Class II histocompatibility antigen, DM alpha chain	Class II	6.82	6.82 × 10^−04^
*SAHA-DAB2*	Class II histocompatibility antigen, DA beta chain	Class II	4.14	3.10 × 10^−04^
*BTN2A2*	butyrophilin subfamily 2 member A2	unrelated	3.49	3.54 × 10^−02^
*NDUFA4L2*	NDUFA4 mitochondrial complex associated like 2	unrelated	2.83	2.72 × 10^−02^

In DFT1.CIITA, several MHC-I heavy chain and accessory genes were strongly induced, depicting a role of CIITA in MHC-I antigen presentation (table 2). These include (i) MHC-I heavy alpha chain genes *SAHA-UA*, *SAHA-UB* and *SAHA-UC*; (ii) *B2M*, which associates with MHC-I alpha chains to form the trimeric structure of MHC-I molecules; (iii) transporter associated with antigen processing 1 (*TAP1*) for peptide transport into the endoplasmic reticulum and (iv) proteasomal subunits *PSMB8* and *PSMB9*.

Next, all significantly up- or downregulated genes were analysed for enriched GO biological processes using DAVID bioinformatics resource. Thresholds *p*-value < 0.05 and FDR < 0.05 were applied to filter out insignificant over-represented GO terms. The most significantly enriched GO biological process in the list of upregulated genes in DFT1.CIITA and DFT2.CIITA was *antigen processing and presentation* (GO:0019882) followed by *immune response* (GO:0006955) (table 4). Both processes were identified in genes of the MHC-I and MHC-II machinery (electronic supplementary material, tables S6 and S7). *Cell adhesion* (GO:0007155) and *cell communication* (GO:0007154) were enriched in genes downregulated in DFT1.CIITA; there were no GO biological processes that were associated with downregulated genes in DFT2.CIITA.

**Table 4.** GO biological processes enriched in differentially expressed genes in DFT1.CIITA and DFT2.CIITA.

GO ID	GO term	count	term size	fold enrichment	*p*-value	FDR
**DFT1.CIITA**						
*upregulated*						
GO:0019882	antigen processing and presentation	8	42	86.09	1.34 × 10^−12^	1.19 × 10^−09^
GO:0006955	immune response	9	518	7.85	4.75 × 10^−06^	2.12 × 10^−03^
* downregulated*						
GO:0007155	cell adhesion	44	719	2.14	2.27 × 10^−06^	4.48 × 10^−03^
GO:0022610	biological adhesion	44	721	2.13	2.44 × 10^−06^	4.48 × 10^−03^
GO:0023052	signalling	113	2746	1.44	4.94 × 10^−06^	6.05 × 10^−03^
GO:0044700	single organism signalling	111	2726	1.42	1.12 × 10^−05^	1.02 × 10^−02^
GO:0007154	cell communication	112	2773	1.41	1.46 × 10^−05^	1.07 × 10^−02^
**DFT2.CIITA**						
*upregulated*						
GO:0019882	antigen processing and presentation	4	42	215.21	9.33 × 10^−08^	3.90 × 10^−05^
GO:0006955	immune response	4	518	17.45	1.87 × 10^−04^	3.91 × 10^−02^

### 3.2. Regulation of MHC-I and MHC-II pathways by Class II transactivator

To further characterize the regulation of MHC-I and MHC-II by CIITA and how it differs from IFNG or NLRC5 stimulation, a heatmap was used to display the relative expression of MHC-I and MHC-II genes, and key accessory proteins between the different treatments. The transcriptome of IFNG-treated DFT2 cells was previously carried out on DFT2 cell line RV (DFT2.WT^RV^) [29] while subsequent experiments on DFT2 cells were performed using DFT2 cell line JV (DFT2.WT). Schwann cell differentiation marker SRY-box 10 (*SOX10*) and neuroepithelial marker nestin (*NES*) were used as internal gene controls, and myelin protein periaxin (*PRX*) was used to discriminate DFT1 cells from DFT2.

**Figure 2.**
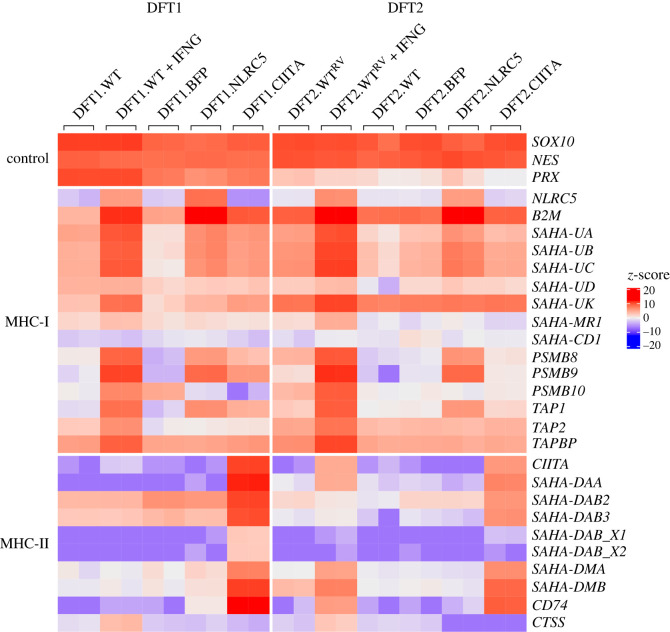
Heatmap showing relative expression of genes involved in MHC-I and MHC-II antigen processing and presentation in WT, IFNG-treated, BFP- (vector control), NLRC5-, and CIITA-expressing DFT1 and DFT2 cells. Z-scores were calculated from log_2_TPM expression values and scaled across each gene (rows). High- and low-relative expressions are represented by red and blue, respectively. Replicates per treatment (*n* = 2) are included in the heatmap.

As described above, CIITA induced the transcription of *B2M;* MHC-I heavy chains *SAHA-UA*, *-UB*, *-UC*; *PSBM8; PSMB9* and *TAP1* in DFT1 cells. There was also an upregulation of non-classical MHC-I *SAHA-UK*, and downregulation of *NLRC5* and proteasomal subunit *PSBM10* in DFT1.CIITA cells (figure 2). Excluding *NLRC5*, genes that were modulated in DFT1.CIITA were synonymously up- or downregulated in DFT1.NLRC5, suggesting similar roles of CIITA to NLRC5 in DFT1 cells. However, the induction of the MHC-I pathway by CIITA was not as strong as NLRC5 despite having similarly high levels of expression in the respective cell lines (figure 2; electronic supplementary material, table S3). IFNG exhibited a wider range in the regulation of genes from the MHC-I pathway compared to NLRC5 and CIITA. Peptide transporter *TAP2* and MHC-I chaperone TAP-binding protein (*TAPBP*) were exclusively upregulated by IFNG in DFT1 and DFT2 cells. Meanwhile, the expression of CIITA in DFT2 cells did not appear to significantly influence any of the MHC-I machinery.

High levels of CIITA transcripts in DFT1.CIITA were correlated with strong induction of all the MHC-II genes, with *SAHA-DAB_X1* and *SAHA-DAB_X2* being the weakest. This was not observed in the other cell lines nor in DFT2.CIITA. CIITA was expressed to a lesser extent in DFT2.CIITA relative to DFT1.CIITA, and all MHC-II genes but *SAHA-DAB_X1* and *SAHA-DAB_X2* were upregulated. The expression of *CIITA*, MHC-II genes and *CD74* was relatively low in DFT1.WT and DFT2.WT cells except for *SAHA-DAB2* and *SAHA-DAB3* in DFT1.WT. There was a moderate increase in CIITA expression after IFNG treatment in DFT1 cells, but it was insufficient to initiate transcription of MHC-II genes or *CD74*. In IFNG-treated DFT2 cells where CIITA was induced to a higher degree, there was only partial activation of the MHC-II gene set (*SAHA-DAA*, *SAHA-DMA* and *SAHA-DMB*), and an upregulation of *CD74*. Interestingly, MHC-II protease cathepsin *CTSS* was only induced with IFNG treatment in DFT1 and DFT2 cells.

### 3.3. MHC-I and MHC-II molecules are upregulated by Class II transactivator in DFT cells

**Figure 3.**
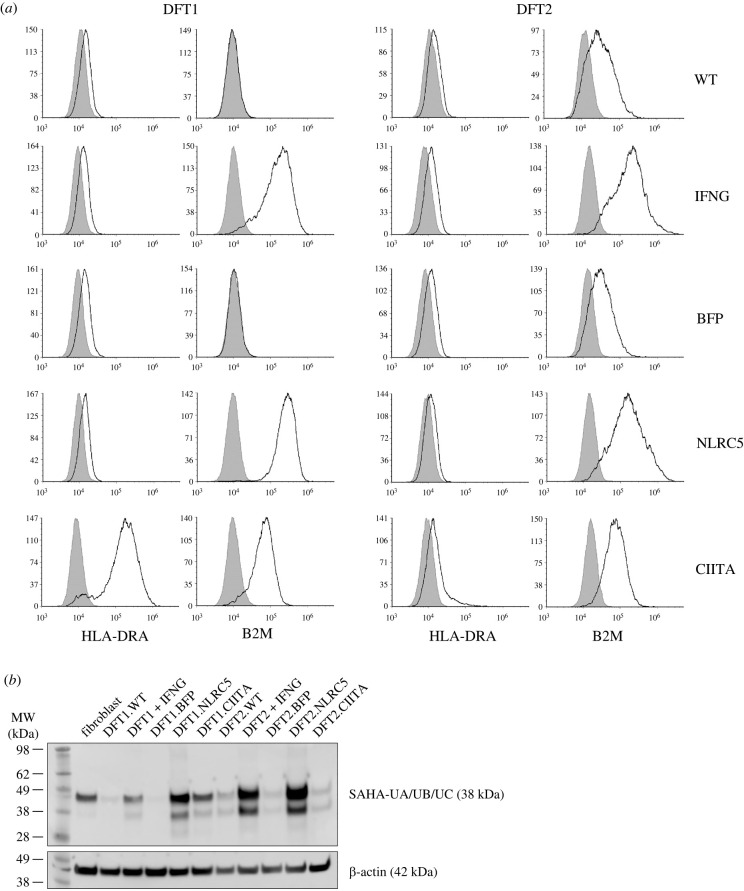
Expression of MHC-II, B2M and MHC-I in DFT1 and DFT2 cell lines. (*a*) WT, IFNG-treated (IFNG), vector-only control (BFP), NLRC5-overexpressing (NLRC5) or CIITA-overexpressing (CIITA) DFT1 and DFT2 cells were analysed by flow cytometry for B2M and MHC-II expression using antibodies against surface devil B2M or intracellular HLA-DR alpha chain (HLA-DRA), respectively (solid line). B2M and MHC-II expressions were overlaid with a secondary antibody-only control (shaded area). The results shown are representative of *n* = 3 replicates/treatment. (*b*) Cell lysate from devil fibroblast, DFT1 and DFT2 cell lines was incubated with an antibody against MHC-I heavy chain genes SAHA-UA/UB/UC for western blot analysis of MHC-I expression. β-actin was included as a loading control. *MW*, molecular weight.

MHC-II (HLA-DRA) protein expression was absent in wild-type (WT) DFT1 and DFT2 cells and in vector-only transfected cells (BFP) but was significantly upregulated in CIITA-expressing DFT1 cells (figure 3*a*). In DFT2 cells, the overexpression of CIITA did not alter median MHC-II expression, or more specifically MHC-II gene loci HLA-DRA. Neither IFNG treatment nor NLRC5 overexpression induced MHC-II protein expression in DFT1 and DFT2 cells. CIITA was capable of restoring surface expression of B2M in DFT1 cells, albeit to a lesser degree than NLRC5 and IFNG stimulation, consistent with the transcriptomic results (figure 3*a*, figure 2). Meanwhile the basal expression of B2M in DFT2 cells was enhanced slightly by CIITA.

In agreement with an increase in surface B2M expression on DFT1.CIITA by flow cytometry, an upregulation of MHC-I heavy chains was detected by western blot compared to wild-type (DFT1.WT) and vector-only cells (DFT1.BFP) (figure 3*b*). IFNG-treated and NLRC5-overexpressing DFT1 and DFT2 cells also expressed elevated levels of MHC-I heavy chains. Although flow cytometry detected an increase in B2M expression on DFT2.CIITA, the expression of MHC-I heavy chains by western blot was similar to DFT2.WT and DFT2.BFP.

### 3.4. Analysis of anti-DFT serum antibody response against Class II transactivator-induced antigens

We have previously shown that MHC-I on DFT1 cells is the predominant antibody target in devils with natural and induced anti-DFT immune response including tumour regressions [8]. Here, we tested if the expression of CIITA in DFT cells could also upregulate antibody targets on DFT cells. Four devils (My, TD4, TD5 and TD6) that developed DFT1 tumours and subsequent serum antibodies (immune) that bound MHC-I were selected for screening against CIITA-expressing DFT1 and DFT2 cells. Serum from each devil prior to DFT1 infection or observable DFT1 tumours (pre-immune) was included to assess the change in antibody levels after DFT1 infection.

**Figure 4.**
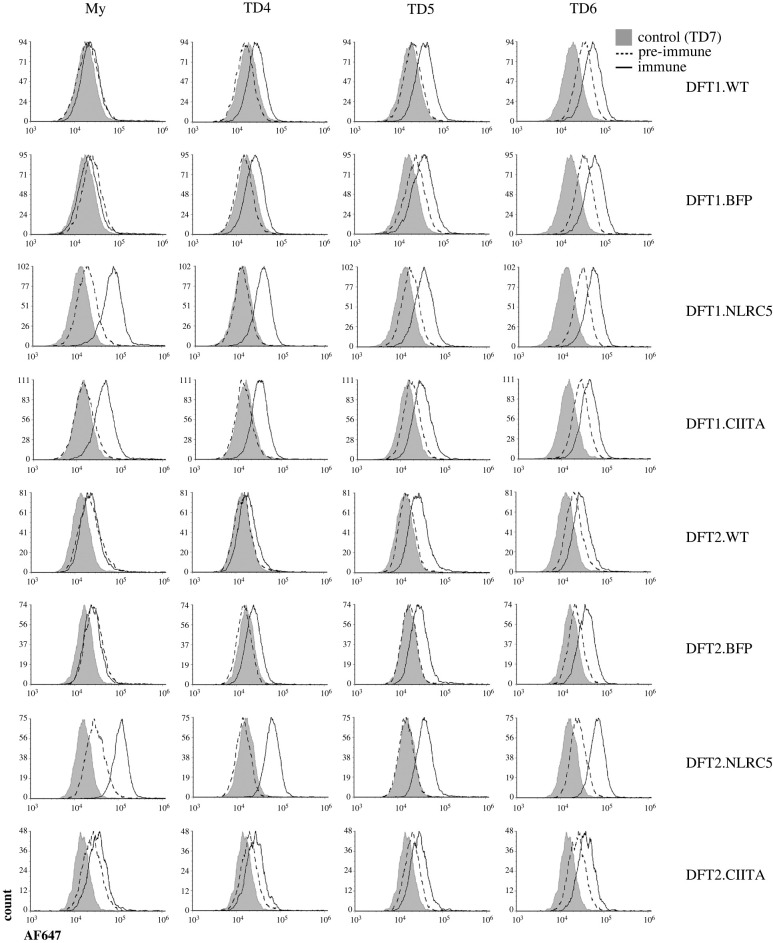
Flow cytometric analysis of serum antibody response towards DFT1 and DFT2 cells overexpressing CIITA. Sera from four devils (My, TD4, TD5, TD6) with antibody responses to MHC-I^+^ DFT1 cells after DFT1 infection (immune) were used. Antibody binding was compared against wild-type (DFT.WT), vector-only (DFT.BFP) and NLRC5-overexpressing cells (DFT.NLRC5). Serums collected prior to infection (pre-immune) and from a non-infected devil (TD7) were included as negative controls. *AF647*, Alexa Fluor 647.

Relative to MHC-I negative DFT1.WT and DFT1.BFP, serum antibodies from all four devils post-DFT1 development generally showed higher binding to DFT1 cells overexpressing NLRC5 (figure 4). Antibody levels against CIITA-expressing DFT1 cells were higher than DFT1.WT and DFT1.BFP in immune sera from My, a captive devil with an immunotherapy-induced DFT1 regression, and TD4, a wild devil with a natural DFT1 regression. Binding of serum antibodies to DFT1.CIITA cells was relatively lower than DFT1.NLRC5. There was no increase in antibody binding towards DFT1.CIITA compared to DFT1.WT and DFT1.BFP from immune sera of devils TD5 and TD6.

Serum from DFT1-infected devils reacted with DFT2 cells but only following NLRC5 overexpression. Serum from My, TD4, TD5 and TD6 all had strong antibody binding to DFT2.NLRC5 which was not observed in the other DFT2 cell lines. This suggests that NLRC5 upregulates similar antigenic target(s) in DFT1 and DFT2 cells.

## 4. Discussion

Clonally transmissible cancers in nature are rare, and yet the Tasmanian devils are affected by two of the only three known naturally occurring transmissible cancers in vertebrates. In a cancer where allogeneity exists between individual host tissues and tumour, allogeneic MHC molecules on tumour cells are important targets for anti-tumour immunity. MHC-I expression on DFT1 cells has been exploited for vaccine development and immunotherapy to enhance anti-DFT immunity via CD8^+^ T cell responses [9]. In this study, we showed that the CIITA can modulate MHC-I and MHC-II antigen processing and presentation pathways in DFT1 and DFT2 cells at the transcriptional level. Surprisingly, the overexpression of CIITA resulted in the upregulation of MHC-I and MHC-II molecules in DFT1 cells but not DFT2 cells.

MHC-II expression is normally confined to a subset of haematopoietic antigen-presenting cells, and DFT1 and DFT2 cells do not typically express MHC-II genes and proteins. We demonstrated the expression of MHC-II proteins in non-haematopoietic DFT1 cells through CIITA-induced upregulation of classical and non-classical MHC-II genes, and the invariant chain *CD74*. The lack of detectable MHC-II proteins in CIITA-expressing DFT2 cells could be due to insufficient expression of MHC-II genes and *CD74* for stable expression of MHC-II molecules. MHC-II expression has been shown to be regulated by CIITA in a qualitative and quantitative manner in different cell types and tissues [56]. The transcription level of MHC-II genes in DFT cells appeared to be relative to CIITA expression. The lower expression of CIITA in CIITA-transfected DFT2 cells compared to CIITA-transfected DFT1 cells could be due to factors related to transgene copy number, genomic stability and integration sites, cellular metabolic state, or selection of low CIITA-expressing cells. Alternatively, the inability to express MHC-II molecules in CIITA-overexpressing DFT2 cells might be due to epigenetic or post-transcriptional regulation, possibly as a consequence of the differentiation state of DFT2 tumours compared to DFT1 [29,57]. A heterozygous non-synonymous mutation (D59N) in transcription factor *RFX5* was also described in DFT2 tumours [6]. RFX5 is a transcription factor of the multi-protein MHC enhanceosome that regulates MHC-I and MHC-II expression [21,58]. Although transcription of MHC-I and MHC-II genes was induced in DFT2 cells following stimulation, the functional impact of this mutation on MHC transcription remains to be explored.

Differential expression of MHC-II allotypes upon CIITA induction, as observed with *SAHA-DAB_X1* and *SAHA-DAB_X2*, that were consistently expressed at lower levels compared to other MHC-II genes, suggests additional regulatory mechanism(s) that control the expression of MHC-II genes beyond that of CIITA. Variations in expression levels of MHC-I and MHC-II genes have been associated with sequence polymorphism in the promoter or 3′ untranslated region of MHC genes, which modulates transcription either epigenetically or non-epigenetically, in addition to post-transcriptional regulation [59–61]. The varying degrees of inducibility and expression of devil MHC-II allotypes could correlate to tissue-specific expression, with functions that differ from classical MHC-II genes.

Consistent with findings from pioneering studies on CIITA function [25,26], CIITA exhibited transcriptional activity over the MHC-I pathway in DFT1 cells that lack MHC-I expression. The ability of CIITA to regulate MHC-I expression is attributed to similarities in the regulatory elements at the proximal promoters of MHC-I and MHC-II genes, and interaction with the same transcription factors of the MHC enhanceosome as NLRC5 [21,24–26,62]. In MHC-I positive DFT2 cells, the overexpression of CIITA resulted in minimal upregulation of MHC-I compared with NLRC5 or IFNG stimulation. The limited CIITA influence on MHC-I expression is commonly observed in cells with high-constitutive levels of MHC-I [25,26]. This illustrates the role of NLRC5 as the primary transactivator for MHC-I expression and a secondary role for CIITA.

Unlike the ubiquitous expression of MHC-I molecules in nucleated cells, MHC-II expression is tightly regulated in a cell type-, differentiation- and stimulus-specific manner through CIITA expression. Evidence for inducibility of MHC-II expression in DFT cells suggests that MHC-II-restricted tumour antigen presentation could occur in the physiological setting under inflammatory conditions that upregulate CIITA. This could provide additional targets for allogeneic and anti-tumour immune responses. In canine transmissible venereal tumour, the tumour regression phase is often associated with the upregulation of MHC-I and MHC-II molecules, mediated by factors such as IFNG from tumour-infiltrating lymphocytes [63,64]. CIITA-overexpressing DFT cells can be exploited for enhancing tumour immunogenicity on a vaccine platform through increased antigen presentation via MHC-I and MHC-II molecules. Our results show that CIITA overexpression in DFT1 cells increases binding of serum antibodies collected from devils of natural and immunotherapy-induced DFT1 regressions.

The capacity to express MHC-II molecules with CIITA expression could stem from the Schwann cell origins of DFT1 and DFT2 cells [29,65]. Schwann cells express MHC-II molecules upon traumatic and inflammatory injury, playing a role in antigen presentation to CD4^+^ T cells to modulate local immune responses [66,67]. Similarly, CIITA-expressing DFT cells have the potential to present MHC-II-restricted tumour antigens to CD4^+^ T cells and potentiate anti-DFT immune responses. Several studies in murine models have demonstrated immune-mediated tumour rejection and/or tumour growth retardation using MHC-II-expressing tumour cell lines, either through CIITA or MHC-II gene transfer [68–73]. These primary responses were also protective against subsequent challenge with parental MHC-II negative tumours. The expression of MHC-II on CIITA-expressing DFT cells can offer insight into the importance of CD4^+^ T cells in the interplay with other immune cells for anti-tumour immunity and allograft rejection. However, the expression of butyrophilin *BTN2A2* by CIITA in DFT1 and DFT2 cells could have an impact on the activation of CD4^+^ T cells as reported in mouse studies [74–76].

In this study, the role of CIITA as a regulator of MHC-II expression was reaffirmed in a non-model immunology research species. We have delineated the regulation of MHC-I and MHC-II pathways by CIITA in Tasmanian devils and transmissible cancers. The ability to induce MHC-II expression in transmissible tumour cells creates an avenue for vaccine and immunotherapeutic strategies to enhance anti-tumour immunity through CD4^+^ T cell help and inform of the importance of MHC-II in anti-tumour and allogeneic immune responses. The relatively simple process we developed for making cell lines that constitutively express NLRC5 and CIITA can be readily adapted for many other species and potentially be used in conjunction with T cell co-stimulatory molecules CD80/CD86 to provide antigen stimulation in *in vitro* assays. This is critical for 99% of species that lack reagents for *in vitro* T cell activation, such as agonistic anti-CD3 and anti-CD28 antibodies.

## Data Availability

Data are available in the electronic supplementary material. Raw RNAseq data have been deposited to the ENA and are available at BioProject # PRJEB45867. The data are provided in the electronic supplementary material [77].
